# Examining size–strength relationships at hippocampal synapses using an ultrastructural measurement of synaptic release probability

**DOI:** 10.1016/j.jsb.2009.10.014

**Published:** 2010-11

**Authors:** Tiago Branco, Vincenzo Marra, Kevin Staras

**Affiliations:** a Department of Neuroscience, Physiology and Pharmacology, University College London, London WC1E 6BT, UK; b Wolfson Institute for Biomedical Research, University College London, London WC1E 6BT, UK; c School of Life Sciences, University of Sussex, Brighton BN1 9QG, UK

**Keywords:** Synapse, FM1-43, Photoconversion, Release probability, Reconstruction, Electron microscopy

## Abstract

Release probability (*p_r_*) is a fundamental presynaptic parameter which is critical in defining synaptic strength. Knowledge of how synapses set and regulate their *p_r_* is a fundamental step in understanding synaptic transmission and communication between neurons. Despite its importance, *p_r_* is difficult to measure directly at single synapses. One important strategy to achieve this has relied on the application of fluorescence-based imaging methods, but this is always limited by the lack of detailed information on the morphological and structural properties of the individual synapses under study, and thus precludes an investigation of the relationship between *p_r_* and synaptic anatomy. Here we outline a powerful methodology based on using FM-styryl dyes, photoconversion and correlative ultrastructural analysis in dissociated hippocampal cultured neurons, which provides both a direct readout of *p_r_* as well as nanoscale detail on synaptic organization and structure. We illustrate the value of this approach by investigating, at the level of individual reconstructed terminals, the relationship between release probability and defined vesicle pools. We show that in our population of synapses, *p_r_* is highly variable, and while it is positively correlated with the number of vesicles docked at the active zone it shows no relationship with the total number of synaptic vesicles. The lack of a direct correlation between total synaptic size and performance in these terminals suggests that factors other than the absolute magnitude of the synapse are the most important determinants of synaptic efficacy.

## 1 Introduction

The likelihood of synaptic vesicle fusion occurring after the arrival of an action potential at a presynaptic bouton is termed synaptic release probability or *p_r_* (Del Castillo and Katz, 1954). This parameter is one of the key determinants of synaptic performance and is therefore of great interest in studies aimed at understanding the fundamentals of neuron–neuron signalling (Maass and Zador, 1999). A complete knowledge of *p_r_* regulation and its determinants will be an important step in fully characterizing signal propagation and processing in neuronal networks (Branco and Staras, 2009). Nonetheless, *p_r_* is relatively difficult to measure directly and this is particularly true in the case of individual synaptic terminals. Thanks to recent advances in molecular and imaging-based technologies, however, new technical approaches have become available over the last 10–15 years to study properties of single synapses (Ryan, 2001). In particular, three main groups of fluorescent probes, the activity-dependent FM-styryl dyes (Betz and Bewick, 1992; Ryan et al., 1993), genetically-encoded synaptic markers such as synaptopHluorin (Miesenbock et al., 1998; Sankaranarayanan and Ryan, 2000), sypHy (Granseth et al., 2006) and vGlut1-pHluorin (Balaji and Ryan, 2007), and more recently fluorescent nanoparticles (Zhang et al., 2009), have been very valuable, yielding important quantitative information on the kinetics of vesicle exocytosis and endocytosis (reviewed in Fernandez-Alfonso and Ryan, 2006). Moreover, some of these probes have been used to provide estimates of release probability at the level of single presynaptic terminals (Aravanis et al., 2003; Branco et al., 2008; Gandhi and Stevens, 2003; Granseth et al., 2006; Murthy et al., 1997; Zakharenko et al., 2001), and have offered important insights into the regulation of this parameter across populations of synapses (Branco et al., 2008; Granseth et al., 2006; Murthy et al., 1997). Although the use of these fluorescent reporters has proved very informative for some applications, the interpretation of data can be difficult, in particular because of the limited spatial resolution offered by conventional light microscopy. As such, unequivocally establishing true correspondence between a fluorescence signal and the presence of an individual synaptic terminal is a major challenge. Moreover, light microscopy provides limited information on many important synapse-specific parameters, such as: number of release sites, measures of synaptic size (synaptic volume, vesicle pool size, active zone area, number of docked vesicles, etc.) and confirmation of pre- and postsynaptic identity. Lack of, or erroneous information of the types outlined here adds error into the characterization of synapses and clouds understanding of the factors which underlie release probability regulation.

One solution to this problem is to use a method where synaptic properties can be read out at ultrastructural resolution. In this case, measures of synaptic performance, such as release probability, could be directly compared with detailed information on synaptic morphology. In this paper, we outline an approach for achieving this based on the use of a fixable form of FM-dye (FM1-43FX). Extracellular FM-dye is readily taken up into synaptic vesicles during endocytosis when synapses are stimulated in the presence of the dye (Betz and Bewick, 1992; Ryan et al., 1993; Fernandez-Alfonso and Ryan, 2004; Granseth et al., 2006; Klingauf et al., 1998). As such, this probe is an excellent marker of vesicles that have fused with the plasma membrane during stimulation, and have been subsequently recycled. Following fixation the dye can be readily photoconverted in the presence of diaminobenzidine into an electron-dense form that is visible in electron micrographs, as demonstrated previously (Darcy et al., 2006a; Harata et al., 2001a; Henkel et al., 1996; Rea et al., 2004; Rizzoli and Betz, 2004; Schikorski and Stevens, 2001). Thus, by counting photoconverted vesicles in serially reconstructed synaptic terminals, it is possible to gain an accurate count of vesicle turnover in response to a defined stimulus – a direct readout of synaptic release probability – and by using correlative methods specifically target this analysis to individual synapses of interest.

Here, we use this approach to examine, with nanoscale resolution, the relationship between anatomically defined vesicle populations and synaptic performance. Previous work using small central synapses has established that many synaptic parameters broadly scale together: for example, synaptic efficacy correlates with the size of the recycling pool and readily releasable pool, as well as other structural variables (Dobrunz and Stevens, 1997; Murthy et al., 1997, 2001; Schikorski and Stevens, 2001; Waters and Smith, 2002). One of the most accessible synaptic parameters for measurement at ultrastructural level is the total vesicle pool size, but it is unclear how this scales with synaptic performance. Here, we examined this directly by measuring *p_r_* and total pool size at fully reconstructed hippocampal synapses. Our findings show that while *p_r_* correlates with the number of vesicles docked at the active zone, a previously established indicator of synaptic strength (Murthy et al., 2001), it is uncorrelated with the total number of vesicles at a terminal. We suggest that this is because the total vesicle pool includes a highly variable fraction of resting vesicles (Fernandez-Alfonso and Ryan, 2008; Harata et al., 2001a) which do not directly contribute to release probability, and thus weakens any direct relationship between release probability and the total number of vesicles observed ultrastructurally. The method outlined in this paper provides an opportunity to directly relate *p_r_* to synaptic parameters that are only amenable to analysis at ultrastructural level, and should be of considerable value in furthering our understanding of the fundamental processes contributing to synaptic function.

## 2 Materials and methods

### 2.1 Cell culture

Dissociated hippocampal neurons were prepared from P0–P1 rats by plating neurons at low density (5000–10,000 per well on 24 well-plate) onto an astrocyte feeder layer growing on poly-d-lysine/collagen coated glass coverslips. The cultures were maintained in Neurobasal-based media (Invitrogen) containing 2 mM Glutamax (Invitrogen), 0.15% glucose, 5% foetal calf serum, 1% penicillin/streptomycin and 10 mM KCl, and used for experiments after 10–15 days *in vitro*. Animal care and use protocols were approved by the Home Office (UK).

### 2.2 FM-dye loading

**Fig. 1:**
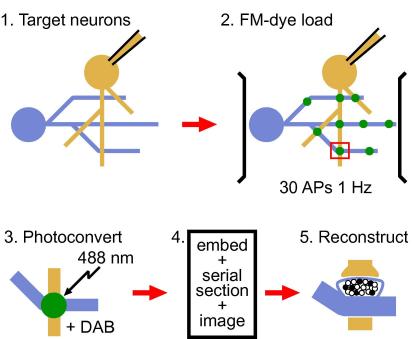
A schematic illustrating the steps in making ultrastructural measurements of *p_r_*. Cultured neurons are targeted (and, for example, filled with fluorescent dye to aid correlative EM) (step 1) before being loaded with FM1-43 using field stimulation (30 APs, 1 Hz) (step 2). After fixation, FM-dye is photoconverted to an electron-dense form in the presence of DAB (step 3) before being embedded, serially sectioned and imaged (step 4). Presynaptic terminals can then be fully reconstructed, permitting a count of the total number of recycled vesicles relative to the defined loading stimulus, and giving a measure of release probability (step 5).

Experiments were carried out in HEPES-buffered solution (125 mM NaCl, 5 mM KCl, 10 mM d-glucose, 10 mM HEPES, 2 mM CaCl_2_, 1 mM MgCl_2_, pH = 7.30) containing CNQX (20 μM) and APV (50 μM) at ∼20 °C. For FM-dye loading (Fig. 1), the extracellular solution included a fixable form of the styryl dye FM1-43 (FM1-43FX, Molecular Probes, 10 μM). Synapses were stimulated in the presence of the dye using field stimulation (30 s, 1 Hz) via parallel platinum wires placed on either side of the imaging chamber. Neurons remained in the dye-solution for a further minute to allow completion of endocytosis and were then washed for 15 min in an extracellular solution containing 0.5 mM CaCl_2_ and 10 mM MgCl_2_ to minimize dye-loss occurring due to spontaneous release. Advasep (Biotium, 1 mM) was included for the first minute of this washing step to aid the removal of surface-bound dye from membranes. FM-dye images were taken using an inverted microscope with a 475/40 nm excitation, 505LP dichroic, 535/45 nm emission filter set. In some cases, target neurons were also filled with Alexa 594 dye via a patch pipette, to provide general information on neuronal morphology and aid correlative analysis.

### 2.3 Photoconversion and correlative ultrastructural analysis

FM-dye labelled neurons were fixed in 2% paraformaldehyde, 2% glutaraldehyde in phosphate-buffered saline (PBS) for 15 min, washed with 100 mM glycine (60 min), then into 100 mM NH_4_Cl (5 min) and subsequently rinsed in fresh PBS. For photoconversion, neurons were preincubated in diaminobenzidine solution (1 mg/ml) for 10 min and then washed into fresh diaminobenzidine solution. The target region was illuminated with 475/40 nm light from a mercury lamp using a 60× 0.9NA objective (15 min). DIC images of the region of interest were obtained at 4×, 10× and 60× and montaged using graphics software (Xara Xtreme). Neurons were then rinsed in PBS, osmicated, stained with tannic acid, dehydrated stepwise in ethanol and embedded in epoxy resin (EPON, TAAB Laboratories) as outlined previously (Darcy et al., 2006a). Serial sections (60 nm) were placed onto formvar-coated slot grids and viewed with a Hitachi-7100 or Phillips EM420 electron microscope. Regions of interest were re-identified using the DIC montage, and images were obtained with a cooled CCD camera (Roper Scientific). Functional presynaptic terminals were identified based on a range of standard criteria including: close apposition to a postsynaptic process, evidence for postsynaptic thickening corresponding to the postsynaptic density, evidence of recycling vesicles and evidence of docked vesicles. We defined docked vesicles as those in a vesicle cluster with membrane in contact (i.e. no resolvable separation) with the plasma membrane of the active zone membrane; the active zone was defined as the region with direct apposition to the postsynaptic density. Quantification of vesicles was performed as outlined previously (Darcy et al., 2006a,b). For *p_r_* measurements we restricted our analysis to clear examples of single synapses containing <800 vesicles with single release sites. 3D reconstructions were made by image alignment, reconstruction and rendering with specialist software (Reconstruct (Fiala, 2005) and Blender, Blender Foundation).

### 2.4 Electrophysiology

**Supplementary Fig. 1:**
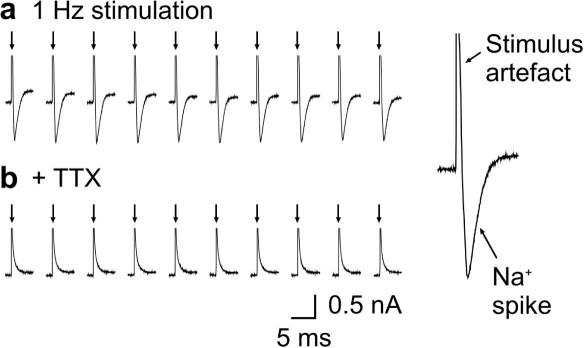
Reliability of field stimulation. (a) Consecutive traces of a whole-cell recording in voltage-clamp mode during field stimulation (1 s interval between traces). Each trace shows an artefactual component arising from the stimulation followed by a prominent unclamped sodium spike. (b) In the presence of TTX, this spike is blocked, confirming that it arises from the activation of voltage-gated Na^+^ channels.

For the experiments shown in Supplementary Fig. 1, whole-cell patch clamp recordings were made from single neurons in the same chamber used for FM-dye loading. Field stimulation parameters were adjusted to reliably trigger fast sodium currents recorded in voltage-clamp mode. This was repeated in a number of experiments in different locations of the coverslip, and only the calibrated areas were used for FM-dye loading experiments. Whole-cell recordings were done as previously described (Branco et al., 2008).

## 3 Results

### 3.1 Experimental approach

Hippocampal neurons grown in culture at relatively low density form well-defined networks. In these networks, individual synapses can be readily labelled with the activity-dependent styryl dye FM1-43 which is taken up into recycling vesicles during endocytosis. Here we outline an approach which uses this vesicle-labelling technique to make detailed measurements of release probability at individual terminals (Fig. 1). The method is based on the principle that stimulating synapses with a defined number of action potentials will evoke a certain level of vesicle release, and that this level is a direct reflection of the release probability at a given synaptic terminal. By assuming tight exocytic–endocytic coupling (Fernandez-Alfonso and Ryan, 2006) we can assess vesicle release by visualizing FM-dye fluorescence accumulated at presynaptic terminals during vesicle endocytosis. To measure this accurately, we photoconvert the fluorescence signal to an electron-dense form so that it can be visualized in ultrastructural detail. In this way, *p_r_* measurements can be made in synapses where detailed structural information is also available.

**Fig. 2:**
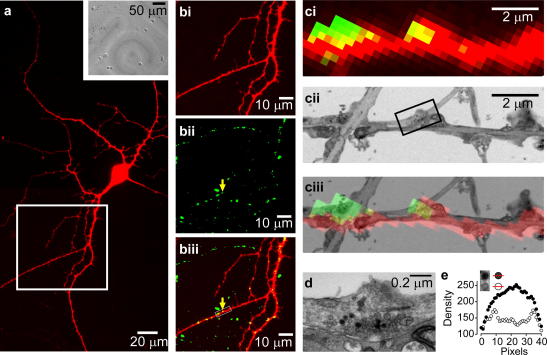
An example of the correlative approach for readout of *p_r_* at a specific presynaptic terminal. (a) Target hippocampal neuron filled with red Alexa dye. Inset shows brightfield image of the region of interest. (bi–biii) Detail of white square region in (a) showing red dye-filled processes (bi), presynaptic terminals labelled with FM1-43 (bii) and a composite overlay (biii). Yellow arrow indicates a putative presynaptic terminal contact site with the red neuron. (c) Detail on contact site in (b). (ci) FM1-43 and red Alexa dye composite. (cii) Equivalent region seen in a low magnification electron micrograph. (ciii) Overlay of (ci) and (cii). (d) Target synapse (indicated by box in (cii)) with photoconverted vesicles (dark lumen) and non-photoconverted vesicles (clear lumen). (e) A photoconverted vesicle can be readily discriminated from a non-photoconverted vesicle with a linescan of optical density.

In our experiments, we chose target neurons of interest (Fig. 2a and bi) and carried out FM1-43 loading by field stimulation of neurons. The loading stimulus was a short train of depolarizing voltage pulses and in pilot experiments we performed whole-cell voltage-clamp recordings from neurons during the stimulation protocol to confirm that each pulse in the stimulus train reliably produced a sodium spike corresponding to an action potential. One example of this experiment is shown in Supplementary Fig. 1. For *p_r_* measurements, we used a stimulus of 30 action potentials at 1 Hz. We chose this minimal stimulation both to limit synaptic depression and the likelihood of re-release of newly-endocytosed vesicles. Pilot experiments based on paired whole-cell recordings suggested that the impact of synaptic depression on our *p_r_* measurement was negligible (data not shown). After loading and washing, synapses were visible as green fluorescent puncta (Fig. 2bii). In some experiments we combined FM-dye loading with cell-filling via a patch pipette so that we could characterize the postsynaptic target of individually-labelled terminals (Fig. 2biii).

To permit ultrastructural quantification of fluorescence signal, we took advantage of the photolabile nature of FM1-43FX and the reactive properties of diaminobenzidine (DAB). Following fixation, a target region in the cultured neurons was identified and illuminated with blue light in the presence of diaminobenzidine for ∼15 min. This photoexcitation of the FM-dye drives diaminobenzidine oxidation and yields an electron-dense product (Henkel et al., 1996). In this way, FM-dye labelled vesicles within the photoexcitation area become selectively darkened. The progression of this photoconversion reaction and its calibration have been discussed previously (Darcy et al., 2006a; Harata et al., 2001a,b; Schikorski and Stevens, 2001).

A key aspect of this approach is the correlation of light microscopy with ultrastructural investigation. This allows post-fixation readout of *p_r_* as well as other structural synaptic properties to be readily linked with dynamic aspects of synaptic function that were previously determined by live fluorescence imaging. This is achieved by compiling a series of brightfield images to form a montage, providing an accurate representation of the relative positions of synapses with respect to the rest of the neuronal architecture (Darcy et al., 2006a,b). After embedding, the region of interest can be re-identified using this ‘map’ and targeted for serial sectioning.

### 3.2 Ultrastructural readout of *p_r_*

**Fig. 3:**
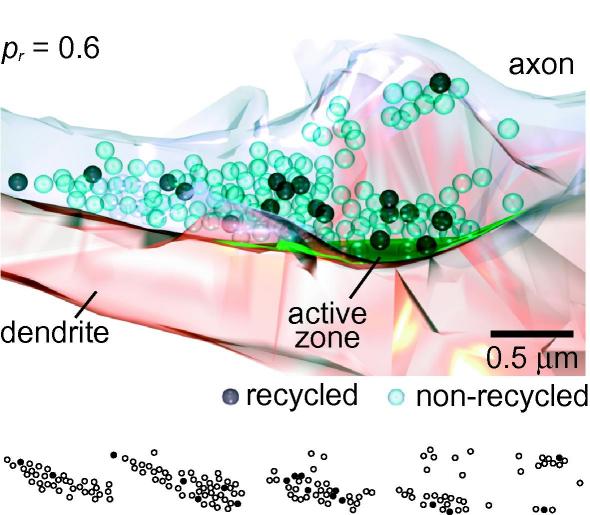
Three-dimensional reconstruction of synapse in Fig. 2 based on a complete section series. Dark vesicles (18) are photoconverted. Non-photoconverted vesicles appear semi-transparent. Release probability is estimated at 0.6. (Bottom) Vesicles in the five sections on which reconstruction is based.

Comparison of both fluorescence images and electron micrographs of the same area reveal a clear correlation between ultrastructurally distinct presynaptic terminals and the location of FM1-43 puncta (Fig. 2c). Moreover, at the electron microscopy level, synapses which were FM-dye positive under the fluorescence microscope are clearly characterized by the presence of small electron-dense vesicles in addition to those with clear lumen (Fig. 2d). These dark vesicles correspond to those that underwent fusion and subsequently endocytosis in response to the loading protocol, during which they took up FM-dye. They can be readily discriminated from unstained vesicles, either visually, or by quantification of, for example, cross-sectional density (Fig. 2e, Darcy et al., 2006b). Thus, by counting the total number of photoconverted vesicles in an individual synaptic terminal we can quantify the number of vesicle exocytosis–endocytosis events that occurred in response to a defined number of action potentials, and directly calculate release probability. In Fig. 3, a target synapse is fully reconstructed revealing 18 electron-dense vesicles, corresponding to a release probability of 0.6. As expected, we were able to confirm that within a synaptic population, FM1-43 signal intensity and ultrastructural measurement of *p_r_* are strongly positively correlated (*R* = 0.84, *P* = 0.0085, *n* = 8, Pearson correlation).

**Fig. 4:**
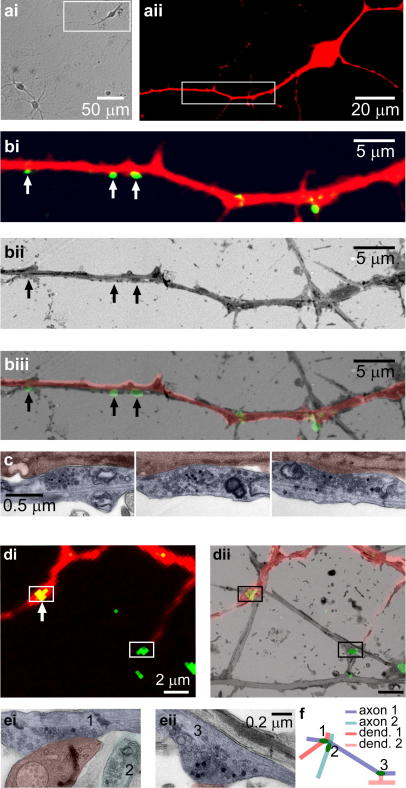
A correlative ultrastructural approach offers additional information to that available by fluorescence. (ai) DIC image of target region, (aii) target neuron filled with red-dye. (b) Detail of region in (a) showing: (bi) overlay of putative postsynaptic neuron (red) and presynaptic contact points (green). (bii) Equivalent region seen in a low magnification electron micrograph. (biii) Composite overlay of (bi) and (bii). (c) Detail of three synapses marked with arrows in (b). Ultrastructural investigation reveals the synapses have shared axonal and dendritic processes. (di) A target region with two clearly-defined FM1-43 puncta and processes of a red dye-filled neuron. (dii) Overlay of fluorescence and low magnification electron micrograph. (ei and eii) Synaptic regions marked by boxes in (d). The ultrastructural detail reveals that presynaptic terminals ‘1’ and ‘3’ share the same axon, but have different target dendritic compartments. *Note:* (di) shows a single punctum on the left (arrow) but the electron micrograph in (ei) confirms the presence of two (‘1’ and ‘2’) terminals in close proximity within this region. (f) Schematic summary of synapse arrangement.

A major advantage of the approach outlined here is that it permits direct comparison between the determined release probability and other structural characteristics of the synapse. This can, for example, include information about the total vesicle number, the spatial organization of vesicles, the number of docked vesicles, the presynaptic or postsynaptic terminal volumes, active zone or postsynaptic density area, number of release sites, the type of synapse, as well as confirmation of postsynaptic apposition. Importantly, it also allows a detailed assessment of synapses and their ‘ownership’ by specific pre and postsynaptic processes. We illustrate this in Fig. 4a–c where correlative ultrastructural microscopy confirms fluorescence data indicating that three target presynaptic terminals share the same axonal and dendritic process. In another example (Fig. 4d–f), the same approach reveals information not apparent from the fluorescence analysis: two distant synapses share a single axon but not a dendritic compartment. Such information is particularly relevant in studies attempting to relate dynamic synaptic properties to the spatial organization of synapses within neuronal networks (e.g. Branco et al., 2008). Additionally, this example illustrates another important point. Although the fluorescence data reports a single FM-dye punctum in the left half of Fig. 4di (arrow) it is clear from the electron micrograph (Fig. 4ei) that this actually corresponds to two functional terminals arising from different axons. The actual arrangement of synapses gained from the ultrastructural information is shown in Fig. 4f.

### 3.3 Examining synaptic size–strength relationships

**Fig. 5:**
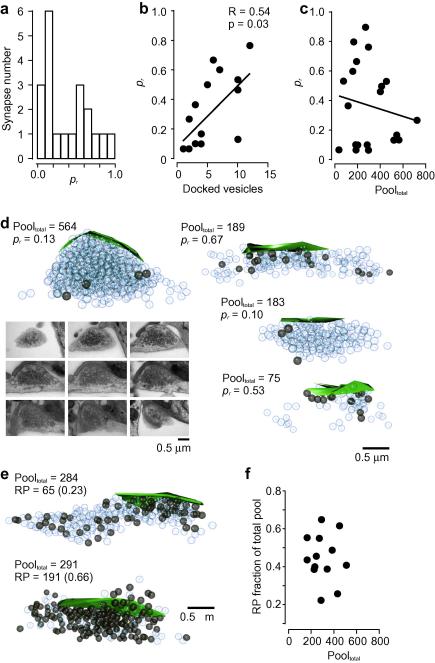
Ultrastructural measurement of *p_r_* and relationship to anatomically defined vesicle populations. (a) Summary *p_r_* distribution plot for synapses (*n* = 20) from three cultures. Values are based on ultrastructural measurements using the approach outlined in this paper. (b) Scatter plot showing *p_r_* against docked vesicle pool size. These parameters are significantly correlated (*R* = 0.54, *P* = 0.03, Pearson correlation). The average number of docked vesicles was 5.8 ± 0.9, similar to the value reported in a previous ultrastructural study in cultured hippocampal neurons (4.6 ± 3.0, Schikorski and Stevens, 1997). (c) Scatter plot for *p_r_* against total vesicle pool size reveals no significant relationship (*R* = −0.16, *P* = 0.50, Pearson correlation). (d) Four examples of reconstructed synapses showing total vesicle count and the number of photoconverted vesicles. bottom left, nine consecutive serial sections for reconstructed synapse on top left. (e)-(f) demonstrating the variability in recycling pool fraction of total pool using FM-dye-photoconversion-EM methods. (e) Two examples of fully reconstructed synapses with very different total vesicle pool recycling fractions. (f) Scatter plot of the recycling fraction of *n* = 13 synapses against their total pool size.

The availability of combined *p_r_* measurements and ultrastructural details at the level of single synapses, offers the opportunity to directly investigate how release probability depends on synaptic anatomy. Here, we used the approach to study the relationship between *p_r_* and two of the most accessible synaptic parameters available in serial electron micrographs: docked vesicle pool size and total vesicle pool size. For simplicity we restricted our analysis to synapses with a single release site. Fig. 5a shows the distribution plot for *p_r_*s drawn from a population of synapses (*n* = 20) from three separate hippocampal cultures. Even with our limited synaptic sample, *p_r_* distribution is broad and skewed with a median of 0.37, which is highly consistent with population *p_r_* measurements obtained previously by us (Branco et al., 2008) and others (Granseth et al., 2006; Murthy et al., 1997; Slutsky et al., 2004) using fluorescence-based approaches, and provides a general validation of this technique. As a further confirmation of our *p_r_* measurement, we next examined the relationship between release probability and the docked vesicle pool size. Previous studies have revealed a positive correlation between *p_r_* and the size of the readily releasable pool determined by fluorescence measurements (Murthy et al., 2001), and between the readily releasable pool size and the number of anatomically docked vesicles, established ultrastructurally (Murthy et al., 2001; Schikorski and Stevens, 2001). This implies that *p_r_* and the number of docked vesicles should be related and our findings here provide direct support for this, revealing a significant positive correlation between these parameters (*R* = 0.54, *P* = 0.03, *n* = 16, Pearson correlation, Fig. 5b). Next, we used our reconstructed synapse population to measure total vesicle pool sizes and compare these to our release probability measurements at the same terminals (Fig. 5c). Surprisingly, our data reveals a clear lack of correlation between these parameters (*R* = −0.16, *P* = 0.50, Pearson correlation). Thus, individual synapses clearly deviate from a simple scaling of total pool size and *p_r_* (Fig. 5d). Why might this be the case? One likely contributing factor is that the number of recycling vesicles at hippocampal synapses, a known correlate of release probability (Murthy et al., 1997, although see Waters and Smith, 2002), is thought to be a highly variable fraction of the total number of vesicles (Fernandez-Alfonso and Ryan, 2008; Harata et al., 2001a). As such, the non-recycling component represents an unknown variable in the relationship between *p_r_* and total pool size, uncoupling the link between the total number of vesicles and synaptic performance. To provide direct support for a variable recycling pool fraction in our cultured neurons, we used the same approach outlined above for our ultrastructural *p_r_* measurements, but this time with a saturating loading stimulus (600 APs, 10 Hz, Ryan and Smith, 1995) to label the total recycling pool. As shown in Fig. 5e and f, we observed a large variability in the total pool fraction of recycling vesicles, with a mean of 0.45 and values ranging from ∼0.2 to ∼0.7. This provides an underlying basis for our observed lack of scaling between the total number of vesicles at a presynaptic terminal and the release probability of the same synapse.

## 4 Discussion

We have outlined a general approach for measuring a key parameter of synaptic strength, release probability, in ultrastructural detail. The method is based on minimal synaptic stimulation combined with activity-dependent labelling of vesicles followed by fluorescence photoconversion and correlative ultrastructural analysis. This technique provides a readout of *p_r_* but also gives detailed correlated information on synaptic morphology. To demonstrate the value of this approach, we have used the ultrastructural readout of *p_r_* to explore the relationship between total pool size and release probability.

Although this is a powerful approach, as with all current methods to estimate *p_r_* (see Branco and Staras, 2009 for discussion), its validity depends on certain assumptions. One issue is the need for good correspondence between the intended defined stimulus and the actual generation of action potentials occurring in target neurons. This is important since the quantitative readout of vesicle recycling, and in turn our estimate of *p_r_*, must be directly related to the number of stimuli applied. In this paper, we used field stimulation, a convenient method for globally activating a population of neurons, and validated the use of this stimulus in pilot experiments by establishing that stimulus delivery was associated with reliable neuronal activation recorded with whole-cell patch clamp. Additionally, we prevented unwanted activity in the neurons by using postsynaptic glutamate receptor blockers to minimize recurrent network activity during stimulation and by washing cells into a low Ca^2+^ extracellular solution after dye-loading to minimize spontaneous transmission.

We also need to be confident that the uptake of FM-dye represents a valid readout of vesicle fusion. This idea is supported by a large body of work illustrating the tight coupling between exocytosis and endocytosis (Fernandez-Alfonso and Ryan, 2006). In our study we used a loading stimulus of 30 APs at 1 Hz to limit synaptic depression and vesicle re-release while maximizing signal available for EM analysis. Pilot experiments based on paired whole-cell recordings suggested that the impact of synaptic depression on our *p_r_* measure was negligible. Also, our work was carried out at room temperature (∼20 °C), where even preferential vesicle reuse would most likely exceed a time of 30 s based on the speed of endocytosis (Fernandez-Alfonso and Ryan, 2004; Granseth and Lagnado, 2008; Klingauf et al., 1998), reacidification (Atluri and Ryan, 2006), mobilization, priming and exocytosis. Nonetheless, we would anticipate that at physiological temperatures, a shortening of the stimulus train (<15 s) would be necessary to correct for the accelerated rate of endocytosis under these conditions (Granseth and Lagnado, 2008). We can not exclude the possibility that our method would underestimate *p_r_* at a synapse utilizing a kiss-and-run mode of release. Although FM-dye photoconversion might well report vesicles undergoing single kiss-and-run events, this would not be true in the case of multiple rounds of this mode of recycling. At present the existence of kiss-and-run in hippocampal synapses is the subject of considerable controversy (e.g. Granseth et al., 2006; Zhang et al., 2009) so it is difficult to definitively establish its impact in our study. Nonetheless, it is worth noting that the *p_r_* values we measure in this paper fall well within the ranges reported by others using different analysis methods (Branco et al., 2008; Granseth et al., 2006; Murthy et al., 1997; Slutsky et al., 2004).

Another consideration is whether the electron-dense vesicular product which arises after the photoconversion reaction, reliably reports the presence of FM-dye before photoconversion. Support for this comes from the substantial body of literature where FM-dye photoconversion product seen at ultrastructural level has been used as a readout for FM-dye uptake (Darcy et al., 2006a; Harata et al., 2001a; Henkel et al., 1996; Rea et al., 2004; Rizzoli and Betz, 2004; Schikorski and Stevens, 2001). Additional support is indicated by the strong correspondence between fluorescence-based estimates of recycling pool sizes (Fernandez-Alfonso and Ryan, 2008; Micheva and Smith, 2005) and estimates based on direct counting of photoconverted vesicles in electron micrographs (Darcy et al., 2006b). Additionally, in the current paper, we found a strong positive correlation between fluorescence-based measures of FM1-43 intensity and ultrastructural estimates of release probability. Also, our quantification of release probability in a synaptic population using ultrastructural analysis is in excellent agreement with examples of *p_r_* distribution derived from estimates using optical recording methods (Branco et al., 2008; Granseth et al., 2006; Murthy et al., 1997; Slutsky et al., 2004), providing a further validation of this method. Furthermore, as discussed above, our measurements of *p_r_* are significantly positively correlated with another proposed indicator of synaptic efficacy, the size of the docked vesicle pool, providing additional support for our experimental approach,

The particular advantage of the approach we outline here is that it provides an opportunity to measure release probability at terminals where we also have a substantially enhanced view of synaptic architecture. One clear application of this is in definitively establishing which terminals within a population share presynaptic and postsynaptic structures. This is particularly important in studies aimed at understanding the spatial regulation of synaptic properties and has recently been applied to determine the organizational principles associated with *p_r_* heterogeneity in a synaptic population in culture (Branco et al., 2008). In the current work we illustrate how this approach can provide important information about connectivity in cultured neurons which is not always apparent from fluorescence-based data. A central assumption of fluorescence-based imaging of presynaptic terminals using FM-dyes is that single punctum correspond to single presynaptic terminals. However, correlative ultrastructural studies reveal that this need not be the case: as we demonstrate here, a discrete fluorescent punctum can actually correspond to multiple synaptic terminals in close proximity. Alternatively, a single fluorescent punctum may correspond to a single synapse, but with multiple release sites. In cultured neurons, >20% of synapses have multiple active zones (Schikorski and Stevens, 1997) so that measurements of *p_r_* based on fluorescence data would only be correct in ∼80% of cases, and provide overestimates in the remainder (Murthy et al., 2001). In trying to understand the organization and regulation of synaptic release probability, these various unknown factors could substantially undermine the value of fluorescence-based analysis. Once again, such uncertainties can be easily resolved at the ultrastructural level.

Here we have used our ultrastructural measurement of *p_r_* to demonstrate that release probability is not correlated with the total number of vesicles at cultured hippocampal synapses. This suggests that while the total vesicle pool size scales together with aspects of synaptic anatomy, such as synaptic volume (Murthy et al., 2001), release probability does not follow the same global scaling principles. This observation has two major implications. The first one is that the total pool size is not a major determinant of release probability. We suggest that the lack of coupling between total pool size and *p_r_* arises because the total number of vesicles includes a variable fraction of ‘resting’ vesicles (Fernandez-Alfonso and Ryan, 2008; Harata et al., 2001a) which do not contribute to activity-driven synaptic performance. Indeed, recent evidence has suggested that this resting pool may have an entirely separate function in supporting spontaneous release at hippocampal synapses (Fredj and Burrone, 2009; Sara et al., 2005). An intriguing question for the future is what determines this variable fraction, and if it can be modulated by individual terminals to change the relative number of recycling synaptic vesicles as a mechanism to adjust synaptic strength. The second implication is that the total number of synaptic vesicles, which is a readily accessible synaptic parameter in ultrastructural investigation, is not a reliable indicator of release probability. On the other hand, the docked vesicle pool size has a significant positive correlation with *p_r_*, and could be used as a putative readout of release probability. This is particularly relevant in studies aimed at understanding network connectivity where only ultrastructural information is available (Denk and Horstmann, 2004).

Although new technologies, such as stimulated emission depletion microscopes, are beginning to overcome the diffraction-limits of conventional light microscopy (Hell, 2007) and thus should allow simultaneous measurements of physiological properties and structure, these methods still do not offer sufficient information to unequivocally define anatomical details of synaptic properties. The correlative approach outlined here provides a relatively straightforward method both to estimate a key parameter of synaptic function and to relate it to specific detailed structural features of single terminals.
